# Enrichment‐Based Duplex PCR for Early Detection of Potentially Virulent *Vibrio harveyi* in Aquaculture

**DOI:** 10.1111/jfd.70224

**Published:** 2026-06-24

**Authors:** Javier Barriga‐Cuartero, Carla Hernández‐Cabanyero, Pablo Ibanyez‐Paya, Carmen Amaro, Belen Fouz

**Affiliations:** ^1^ University Institute for Biotechnology and Biomedicine Research (BIOTECMED) Universitat de València Burjassot Spain

**Keywords:** alkaline peptone water enrichment, aquaculture, diagnostic performance, duplex PCR, *fpcrp* gene, *Vibrio harveyi*, virulence‐associated plasmid

## Abstract

*Vibrio harveyi* is an important bacterial pathogen in marine aquaculture, responsible for outbreaks affecting a wide range of fish species. Early detection of potentially virulent isolates in fish and environmental samples is essential for effective disease prevention and management, particularly under field conditions where the pathogen may be present at low abundance. In this study, we developed and evaluated the analytical and diagnostic performance of a two‐step detection protocol that combines a short alkaline peptone water (APW‐1) enrichment step with a duplex PCR assay targeting the species‐specific *toxR* gene and the plasmid‐associated *fpcrp* gene, a putative marker linked to increased virulence in some fish‐pathogenic vibrios. This marker was detected in a diverse set of publicly available genomes from virulent *V. harveyi* isolates as well as in virulent isolates used in this study. This integrated approach enables reliable detection of *V. harveyi* in complex biological and environmental matrices while simultaneously providing virulence‐associated screening. The duplex PCR showed high analytical specificity for *V. harveyi* based on *toxR* amplification when tested against a panel of closely related *Vibrio* species and allowed the identification of *V. harveyi* isolates carrying the putative virulence‐associated marker. Without enrichment, the limit of detection was approximately 10^5^ CFU/g or CFU/mL in fish tissues and water samples, whereas incorporation of an 8 h APW‐1 enrichment step improved the analytical sensitivity to around 10^2^ CFU/g or CFU/mL. The diagnostic performance of the complete two‐step protocol was evaluated using experimentally spiked samples and field samples from Mediterranean aquaculture settings, including fish tissues and aquatic environments. Overall, this enrichment‐duplex PCR assay provides a rapid, sensitive and practical tool for the early detection and risk‐oriented monitoring of potentially virulent *V. harveyi* isolates in aquaculture environments, supporting surveillance programmes and control strategies.

## 1 Introduction

*Vibrio harveyi* is a Gram‐negative, halophilic bacterium widely distributed in marine environments and currently recognized as a major bacterial pathogen in marine aquaculture (Austin and Zhang 2006; Zhang et al. 2018, 2020). This species is responsible for vibriosis outbreaks affecting a broad range of marine vertebrate and invertebrate hosts, a feature that reflects its high genetic variability and remarkable capacity for ecological and host adaptation (Amaro et al. 2020; Austin and Zhang 2006; Mougin et al. 2021; Xu et al. 2017; Zhang et al. 2020). This genetic diversity has been associated with the emergence of highly virulent clones, and with the ability of *V. harveyi* to cause recurrent disease outbreaks in aquaculture facilities worldwide, often resulting in high mortality rates and significant economic losses (Chatterjee and Haldar 2012; Darshanee Ruwandeepika et al. 2012; Haldar et al. 2010; Travers et al. 2009; Triga et al. 2024; Wei et al. 2019). In recent years, the incidence and severity of *V. harveyi* ‐associated diseases have increased, a trend that has been linked to environmental changes in marine ecosystems, particularly rising seawater temperatures driven by global warming (Amaro et al. 2020; Montánchez and Kaberdin 2020; Travers et al. 2009).

Although *V. harveyi* primarily causes diseases in marine organisms, it has also been reported as a rare opportunistic human pathogen (Austin 2010). To date, only five cases have been documented, mainly involving wound infections or bacteraemia following marine exposure or injuries, reported between 1989 and 2020 in the United States, France, Australia and Spain (Brehm et al. 2020; Del Gigia‐Aguirre et al. 2017; Hundenborn et al. 2013; Pavia et al. 1989; Wilkins et al. 2008).

Phylogenetically, whole‐genome sequence–based analyses place *V. harveyi* within the Harveyi clade (Urbanczyk et al. 2013), a core group of *Vibrio* species known for their pathogenicity in marine fish, crustaceans and molluscs (Austin and Zhang 2006; Thompson et al. 2005). This clade has been widely used as model organisms in studies of bioluminescence, quorum sensing and biofilm formation (Dunlap 2009; Dunlap and Urbanczyk 2013; Henke and Bassler 2004; Yildiz and Visick 2009).

Pathogenic *V. harveyi* strains are not serologically homogeneous, and at least three dominant serotypes have been reported to circulate among aquaculture facilities in the Mediterranean basin (Pavlinec et al. 2022). This serological diversity represents a significant limitation for disease control, as current vaccination strategies may fail to confer broad protection, thereby necessitating continued serotyping and epidemiological monitoring (Hernández‐Cabanyero et al. 2025). In this context, increasing evidence indicates that the enhanced virulence of certain *Vibrio* spp. strains is associated with acquisition of virulence plasmids within aquaculture environments (Amaro et al. 2020; Carmona‐Salido et al. 2021). Notably, some *V. harveyi* isolated from diseased European sea bass ( *Dicentrarchus labrax* Linnaeus, 1758) have been shown to harbour a putative virulence plasmid (pVH2) with a high degree of homology to the pVir plasmid (formerly pVvBt2) of *V. vulnificus* pathovar (pv.) *piscis* (Amaro et al. 2020). In *V. vulnificus* , this plasmid confers resistance to fish innate immunity and has been demonstrated to be species‐specific and functionally linked to pathogenicity (Carmona‐Salido et al. 2021; Lee et al. 2008). Among the conserved genes shared by these plasmids, *fpcrp* (fish phagocytosis and complement resistance protein) exhibits near‐complete sequence conservation (approaching 100% identity) and encodes an outer membrane lipoprotein that enables evasion of fish complement‐mediated killing and subsequent phagocytic clearance (Hernández‐Cabanyero et al. 2019; Lee et al. 2008). Other authors have recently reported that selected virulent isolates of *V. harveyi* harbour multiple plasmids, some acting as drivers of virulence in barramundi ( *Lates calcarifer* ) (Sullivan et al. 2025). Current methods for the identification of *V. harveyi* include phenotypic approaches, such as biochemical tests and ELISA (Alavandi et al. 2006), as well as molecular approaches, including multilocus sequence analysis (MLSA) (Cano‐Gomez et al. 2011) and MALDI‐TOF mass spectrometry (Dieckmann et al. 2010). However, biochemical identification may yield inconclusive results (Jiang et al. 2013), MLSA is costly and time consuming (Thompson et al. 2005, 2007) and MALDI‐TOF instrumentation represents a substantial capital investment for many laboratories (Tran et al. 2015). In addition, none of these methods allows discrimination between virulent and non‐virulent *V. harveyi* strains. Therefore, the development of a rapid, sensitive and cost‐effective method to specifically detect *V. harveyi* isolates and, at the same time, to discriminate those with increased virulence potential is highly desirable for the aquaculture industry.

Based on the established role of virulence plasmids in fish‐pathogenic vibrios and the evidence of homologous plasmid‐borne genes in *V. harveyi* , we hypothesised that the *fpcrp* gene is associated with increased virulence potential in certain *V. harveyi* isolates. To test this hypothesis, we developed a two‐step detection protocol combining a species‐specific marker (*toxR*) (Pang et al. 2006) with *fpcrp* as a putative virulence‐associated marker for risk‐oriented detection. A secondary objective was to enhance analytical sensitivity by incorporating a short enrichment step and to assess the preliminary diagnostic performance of the complete two‐step protocol using both experimentally inoculated biological matrices and field samples from aquaculture‐related aquatic environments.

## 2 Material and Methods

### 2.1 Bacterial Strains and Growth Conditions

All *Vibrio* strains used in this study and their main characteristics are listed in Table 1. As clinical Mediterranean isolates of *V. harveyi* are serologically heterogeneous (Amaro et al. 2020; Pavlinec et al. 2022), some belonging to the major serotypes of epidemiological importance (A, B and C) were selected, together with an environmental strain isolated from brackish water. The remaining *Vibrio* species ( *V. vulnificus* , *V. parahaemolyticus* , *V. anguillarum* , *V. alginolyticus* , *V. campbellii* , *V. rotiferianus* and *V. cholerae* ) were selected for thriving in similar environments and for their phylogenomic proximity with *V. harveyi* (Ashok Kumar et al. 2020).

**TABLE 1:** Main characteristics of *Vibrio* strains used in this study.

*Vibrio* strain	Isolation data		Subtyping		
	Source	Country, year	Serotype^a^	*toxR* PCR	*fpcrp* PCR
*V. harveyi* C2	Diseased sea bass	Spain, 2020	C	+	+
*V. harveyi* SK77	Diseased sea bass	Spain, 2015	A	+	+
*V. harveyi* 203	Diseased sea bass	Spain, 2018	B	+	+
*V. harveyi* M1	Brackish water	Spain, 2023	b	+	−
*V. vulnificus* pathovar *piscis* CECT 4999	Diseased eel	Spain, 1993		−	+
*V. parahaemolyticus* TW2^c^	Diseased shrimp	Taiwan, unknown		−	−
*V. cholerae* A8	Diseased tilapia	Spain, 2019		−	−
*V. alginolyticus* Lub1	Healthy sea bass	Spain, 2022		−	−
*V. campbellii* CAIM 519	Seawater	USA, 1971		−	−
*V. anguillarum* Lab1	Diseased sea bass	Spain, 2010		−	−
*V. rotiferianus* CAIM 577	*Brachionus plicatilis* culture	Belgium, 2003		−	−

Abbreviations: CAIM, collection of aquatic important microorganisms; CECT, Spanish collection of type cultures.

^a^ Serology determination from Hernández‐Cabanyero et al. (2025) and Pavlinec et al. (2022).

^b^ Strain M1 did not agglutinate with antisera for *V. harveyi* serotypes A, B or C.

^c^ Strain supplied by Dr. Lien‐I Hor (National Cheng‐Kung University, Tainan, ROC).

Strains were routinely grown on tryptic soy agar supplemented with 5 g/L NaCl (TSA‐1) plates or in Luria‐Bertani broth plus 5 g/L NaCl (LB‐1) at 28°C for 18–24 h with shaking (100 rpm). Strains were stored at −80°C in LB‐1 containing 20% (v/v) glycerol. Thiosulfate Citrate Bile Salts Sucrose (TCBS) agar was used to isolate *V. harveyi* from environmental samples (Austin and Austin 2016), followed by purification by streaking onto TSA‐1 plates.

### 2.2 Fish and Water Sampling and *V. harveyi* Isolation

Given that European sea bass ( *D. labrax* ) is a major host for *V. harveyi* and a key aquaculture species in the Mediterranean basin, this species was specifically selected for evaluation of the diagnostic performance of a new protocol (Pujalte et al. 2003; Triga et al. 2024). Two independent fish samplings were conducted in a local farm in June and September 2022 using healthy specimens previously vaccinated in accordance with standard farm practices. At the time of sampling, water temperature and salinity were recorded as 24°C and 3.7% for the first sampling, and 26°C and 3.7% for the second sampling, respectively. Fish were necropsied following euthanasia by using 3‐aminobenzoic acid ethyl ester (MS‐222) (Sigma) over‐exposure (100 μg/mL), and all efforts were made to minimise suffering. Skin mucus, gills and blood were collected aseptically. Tissues from three individuals were pooled by matrix and processed as described below.

Environmental water samples were collected in July 2023 from La Albufera (Valencia, Spain), a shallow coastal lagoon located on the Mediterranean coast, and from a nearby beach (Figures S1 and S2, Table S1). During sampling, water temperature ranged between 29°C and 30°C. Salinity varied according to the sampling site, reaching 0.2% in the lagoon and 0.5% at the beach. Water samples (50 mL) were collected in sterile containers and processed as described in subsequent sections.

Fish and water samples were streaked onto TCBS agar to isolate *V. harveyi* . Presumptive *V. harveyi* colonies were selected based on typical colony morphology and sucrose fermentation profile on TCBS agar, mainly yellow colonies consistent with sucrose fermentation, and were subsequently purified on TSA‐1 and confirmed by duplex PCR.

### 2.3 Preparation of Inoculated Biological Matrix

Spleens from healthy European sea bass specimens (Section 2.2) were dissected aseptically and stored at −80°C until use (Figure S1). Thawed splenic tissue slices (approximately 0.1 g) were inoculated with 100 μL of 10‐fold serial dilutions of *V. harveyi* strain C2 prepared in 1× phosphate‐buffered saline (PBS, pH 7), targeting final concentrations ranging from 10^0^ to 10^8^ CFU/g of tissue.

The inoculated biological matrix was immediately homogenised using sterile Eppendorf micropestles. Aliquots were subsequently processed either directly for DNA extraction or subjected to an enrichment step as described below. The number of viable bacterial cells in the inoculum was determined using the drop plate count method (Reed and Reed 1948).

### 2.4 Enrichment Procedure

To improve the sensitivity of *V. harveyi* detection, an enrichment step was included prior to DNA extraction. Homogenised biological matrices and environmental water samples were diluted 1:10 (v/v) or 1:1 (v/v), respectively, in alkaline peptone water supplemented with 1% NaCl (APW‐1, pH 8.6). Samples were incubated at 28°C for 8 h with shaking at 100 rpm to promote the selective growth of *Vibrio* spp.

### 2.5 Bacterial DNA Extraction

Genomic DNA was extracted from bacterial cultures, inoculated biological matrices and enriched samples using two different methods to compare speed and DNA quality: a commercial purification kit specifically designed for fish tissue samples and a modified boiling technique (Roig et al. 2022). Briefly, for boiling method, 1 mL aliquots of samples were centrifuged at 12,000 rpm for 4 min at 4°C and the resulting bacterial pellets were resuspended in 100 μL of PBS. To induce bacterial lysis, the suspensions were boiled at 100°C for 5 min in a heat block, immediately chilled on ice for 5 min, and then centrifuged again at 12,000 rpm for 4 min at 4°C using a Gyrozen 1730R centrifuge (Controltecnica, Spain). The supernatant, containing the extracted DNA, was stored at −20°C and used as a template for subsequent PCR amplification.

*V. harveyi* strain C2 was included as a positive control in all PCR assays.

For the commercial extraction method, thawed splenic tissue samples, either intact or experimentally inoculated, were homogenised as described above. Genomic DNA was then extracted according to the manufacturer's instructions using the Genomic DNA Purification Kit (Thermo Fisher Scientific, #K0512). After the final wash step, DNA was eluted in 50 μL of sterile deionised water.

For the modified boiling technique, pure cultures of all *Vibrio* strains were grown in LB‐1 medium for 18 h at 28°C. Cultures were subjected to 10‐fold serial dilutions and centrifuged at 12,000 rpm for 4 min at 4°C prior to DNA extraction. Mixtures of *Vibrio* cultures were also prepared and processed using the same procedure. In addition, DNA from homogenised biological matrices and enriched samples was extracted using the boiling technique.

DNA purity and quality were assessed spectrophotometrically by measuring the *A*_260_/*A*_280_ ratio using a NanoDrop ND1000 spectrophotometer (Thermo Fisher Scientific, Spain). DNA samples were stored at −20°C and used as templates for subsequent PCR amplification.

### 2.6 In Silico Analysis: Selection of the *fpcrp* Gene as a Putative Virulence‐Associated Marker

The genomic dataset used for this analysis comprised *V. harveyi* sequences obtained from our laboratory strain collection, as well as publicly available complete and draft genomes retrieved from the NCBI GenBank database (Table 2). Screening was performed using local BLASTn (Altschul et al. 1990). Significant hits were filtered based on a threshold of > 90% query coverage and > 90% sequence identity. Homologous sequences were extracted according to their genomic coordinates using SnapGene software (www.snapgene.com). Verification of the complete open reading frames (ORFs) and in silico translation were performed using NCBI ORFfinder (Rombel et al. 2002). Final pairwise sequence alignments at both nucleotide and amino acid levels were executed via BLASTn and the EMBOSS Needle global alignment tool (Rice et al. 2000), respectively, to determine the exact number of mismatches, percentage of identity and percentage of similarity.

**TABLE 2:** In silico screening and sequence conservation analysis of the *fpcrp* gene across publicly available *V. harveyi* genomes compared to the *V. vulnificus* reference sequence.

Strain ID	Source/Host	*fpcrp* presence	Nucleotide identity (%)	Accession no.
C2^a^	Diseased sea bass	+	99.142	N/A
203^a^	Diseased sea bass	+	99.070	N/A
SK77^a^	Diseased sea bass	+	99.070	N/A
VhP1	Diseased sea bass	+	99.070	SAMN21546524
SS1	Diseased amberjack	+	99.142	SAMN25050340
A2	Diseased sea bass	+	99.142	SAMN18310300
150/16	Diseased sea bass	+	99.142	SAMN18310301
94/17	Diseased sea bass	+	99.070	SAMN18310299
Lcv6	Diseased yellow croaker	+	99.000	GCA_042691635.1
N8T11	Diseased yellow croaker	+	99.070	GCA_039544385.1
NH‐LM1	Diseased sea bass	+	99.070	GCA_037997145.1
R2B^a^	Healthy sea bass	−	N/A	N/A
PujA^a^	Brackish water	−	N/A	N/A
M1^a^	Brackish water	−	N/A	N/A
WXL538	Seawater	−	N/A	GCA_009184745.1
H2O_078	Reef seawater	−	N/A	SAMN06921171
FDAARGOS_107	Shark (mouth)	−	N/A	GCA_001558435.2
FDAARGOS_109	Dead amphipod	−	N/A	GCA_001471575.2
20MO08SH02_V2	Shrimp (seafood sample)	−	N/A	SAMN16134147
GAN 1807	Greater amberjack (eye)	−	N/A	SAMD00166353
CDDH_034	Healthy elkhorn coral	−	N/A	SAMN06921108
CDPX_247	Healthy elkhorn coral	−	N/A	SAMN06921133
CH_153	Healthy elkhorn coral	−	N/A	GCA_028991095.1

Abbreviation: N/A, not available, not applicable.

^a^ Spanish *Vibrio harveyi* strain used in this study.

### 2.7 Duplex PCR

A novel duplex PCR method was developed for the specific identification of *V. harveyi* strains and the simultaneous identification of isolates carrying the virulence‐associated *fpcrp* gene. The assay combines two primer sets targeting *toxR* (Pang et al. 2006) and the plasmid‐associated *fpcrp* gene (Table 3). Primers targeting *fpcrp* were designed using the Primer‐BLAST software tool (Ye et al. 2012). The target sequence was obtained from the pVH2 plasmid (NCBI, accession no. NZ_CP150469.1) and primer design parameters were optimised to generate an amplicon clearly distinguishable in size from the *toxR* fragment. To ensure high specificity within marine bacterial communities, the in silico search was restricted to members of the Vibrionaceae family. All primers were synthesised by Integrated DNA Technologies (IDT, Belgium), reconstituted and diluted to a final working concentration of 10 μM.

**TABLE 3:** Primers and amplicon sizes used in the duplex PCR assay.

Primer	Sequence (5′–3′)	Amplicon size (bp)	References
toxR_Fw	GAAGCAGCACTCACCGAT	382	Pang et al. (2006)
toxR_Rv	GGTGAAGACTCATCAGCA		
fpcrp_Fw	AGGTAAGAAGGGAGGAGTTT	497	This study
fpcrp_Rv	TCAAGGTTTTTCCGCTAACT		

PCR reactions were performed in a final volume of 25 μL using the Speedy Supreme NZYTaq 2× green Master Mix (NZYTech), following the manufacturer's recommendations. Each reaction mixture contained 12.5 μL of Master Mix, 0.5 μL of each primer (forward and reverse for both targets), 1 μL of DNA template and nuclease‐free water to reach final volume. Amplifications were carried out in a MiniAmp Thermal Cycler (Thermo Fisher Scientific) under the following conditions: an initial denaturation step at 95°C for 3 min, followed by 30 cycles of denaturation at 95°C for 30 s, annealing at 55°C for 5 s and extension at 72°C for 5 s, with a final extension at 72°C for 5 min.

Each PCR run included a negative control containing molecular‐grade water and a positive control consisting of purified genomic DNA from *V. harveyi* strain C2.

Amplification products were resolved by electrophoresis on 1.8% (w/v) agarose gels containing RedSafe nucleic acid staining solution (Intron Biotechnology) at 100 V for 30 min. A GeneRuler 1 kb DNA ladder (Thermo Fisher Scientific), mixed with TriTrack DNA loading dye, was used as a molecular size marker. Gels were visualised using a Microtek MFS‐300L gel documentation system.

#### 2.7.1 Analytical Specificity

The analytical specificity of the duplex PCR assay was evaluated using genomic DNA from *V. harveyi* isolates representing the dominant serotypes of epidemiological relevance, as well as from closely related non‐target *Vibrio* species that coexist in similar marine environments (Table 1). Each primer set was tested individually and in combination against DNA from each *Vibrio* species and against mixtures of genomic DNA from different *Vibrio* spp.

The *fpcrp* marker was interpreted only in conjunction with *toxR* amplification. Accordingly, detection of *V. harveyi* was based on amplification of the species‐specific *toxR* gene, whereas amplification of *fpcrp* in *toxR*‐positive samples was used to identify isolates carrying the putative virulence‐associated marker and, therefore, with increased virulence potential.

#### 2.7.2 Analytical Sensitivity: Limit of Detection (LOD)

The limit of detection (LOD) was defined as the lowest bacterial concentration consistently yielding positive amplification of both target fragments in all replicates. LOD determination experiments were performed in triplicate, and results were expressed as CFU/mL or CFU/g, depending on the sample matrix.

LOD assays were conducted using 10‐fold serial dilutions of *V. harveyi* strain C2, either processed directly or following the enrichment step described in Section 2.4. Moreover, splenic sea bass tissue samples, artificially inoculated (described in Section 2.3), were also processed either directly or following the enrichment procedure outlined in Section 2.4. In both cases, samples were subsequently analysed by duplex PCR to evaluate detection performance across a range of bacterial loads.

#### 2.7.3 Evaluation of Diagnostic Performance

The diagnostic performance of the two‐step duplex PCR protocol was evaluated using field samples, providing a preliminary estimate of its applicability under aquaculture‐relevant conditions.

Fish tissue samples (skin mucus, gills and blood) and environmental water samples collected as described in Section 2.2 were processed according to the two‐step protocol. Fish tissues were pooled by matrix and homogenised prior to enrichment, while water samples were subjected directly to enrichment. Following enrichment, DNA was extracted from all samples and analysed by duplex PCR targeting *toxR* and *fpcrp*.

In parallel, enriched samples were subjected to conventional microbiological analysis by direct plating onto TCBS agar media to support molecular detection results. To this end, enriched samples were streaked onto TCBS agar plates and incubated at 28°C for 24 h. Presumptive *V. harveyi* colonies were selected based on typical colony morphology and sucrose fermentation profile (yellow colonies, sucrose‐positive). Selected colonies were purified by streaking onto TSA‐1 plates and incubated under the same conditions. Pure isolates were subsequently subjected to molecular identification and virulence typing using the duplex PCR assay described above, targeting the species‐specific *toxR* gene and the putative virulence‐associated *fpcrp* marker. This approach allowed confirmation of species identity and discrimination between potentially virulent (*fpcrp*‐positive) and non‐virulent (*fpcrp*‐negative) *V. harveyi* isolates recovered from field samples.

## 3 Results

### 3.1 Development of the New Duplex PCR

The putative virulence‐associated marker selected for this study, the *fpcrp* gene, was detected in a diverse set of publicly available ‘virulent’ *V. harveyi* genomes as well as in those of virulent isolates of our laboratory collection (Table 2). Furthermore, it was found to be absent in a significant number of strains isolated from the environment included in our dataset and published studies (Table 2). Our genomic survey using BLAST against the panel of *V. harveyi* genomes revealed a high level of sequence conservation. Specifically, the 1398 bp coding sequence (CDS) extracted from positive *V. harveyi* strains exhibited more than 99% nucleotide identity (with only 12–13 mismatches) and a 98.3% amino acid identity (98.9% amino acid similarity) when aligned par‐to‐par via EMBOSS Needle against the *V. vulnificus* reference protein.

The analytical specificity of the duplex PCR assay was evaluated using a panel of *V. harveyi* strains and closely related *Vibrio* species selected based on phylogenomic proximity and ecological overlap (Table 1). Amplification of the species‐specific *toxR* marker was exclusively observed in *V. harveyi* strains, while no amplification was detected for any of the non‐target *Vibrio* species tested, either individually or in mixed DNA samples (Figure 1). These results confirm the high specificity of the *toxR*‐based detection and exclude cross‐reactivity with other members of the Vibrionaceae commonly present in marine and aquaculture environments.

**FIGURE 1:**
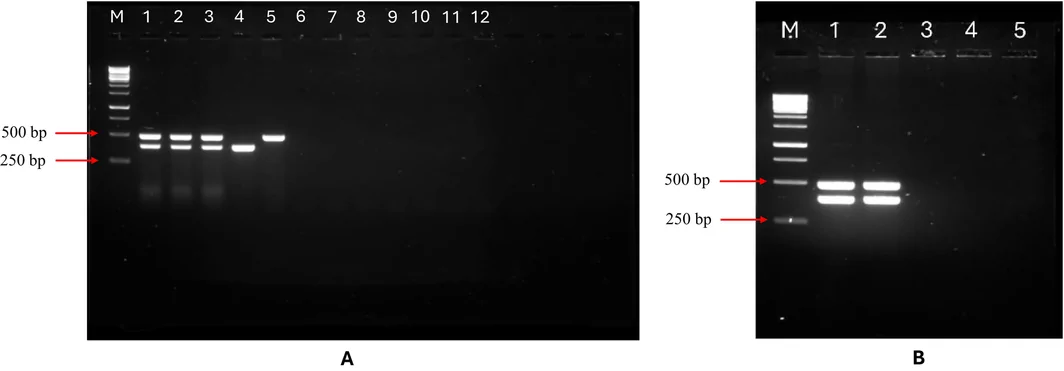
Analytical specificity of the duplex PCR targeting potentially pathogenic *Vibrio harveyi* . Agarose gel electrophoresis of the PCR products obtained from DNA samples of pure cultures of *Vibrio* spp. Amplicons of the *V. harveyi* specific *toxR* (382 bp) and *fpcrp* (497 bp) genes can be visualised when using DNA from single strains (A) or mixtures of DNA from all strains (B). From left to right: (A) 1 kb molecular weight DNA ladder (M), *V. harveyi* C2 (1), *V. harveyi* SK77 (2), *V. harveyi* 203 (3), *V. harveyi* M1 (4), *V. vulnificus* pathovar *piscis* CECT 4999 (5), *V. cholerae* 8A (6), *V. alginolyticus* Lub1 (7), *V. parahaemolyticus* TW2 (8), *V. anguillarum* Lab1 (9), *V. campbellii* CAIM 519 (10), *V. rotiferianus* CAIM 577 (11), negative control (12). (B) M, mixture of DNA from all *Vibrio* strains tested in A including *V. harveyi* and *V. vulnificus* (1 and 2) or not (3 and 4), negative control (5).

The virulence‐associated marker *fpcrp* was detected only in a subset of *V. harveyi* strains known to harbour virulence plasmids, as well as in *V. vulnificus* pv. *piscis*, in agreement with previous reports (Carmona‐Salido et al. 2021; Lee et al. 2008). Importantly, amplification of *fpcrp* was interpreted exclusively in samples that were positive for *toxR*, ensuring that the detection of this marker was unambiguously attributed to *V. harveyi* and preventing misinterpretation in mixed bacterial populations (Figure 1).

The analytical sensitivity of the duplex PCR was first assessed without an enrichment step using serial dilutions of *V. harveyi* strain C2 in water and fish tissue matrices. Under these conditions, LOD was approximately 10^5^ CFU/mL for liquid samples and 10^5^ CFU/g for fish tissue samples, based on consistent amplification of both target fragments in all replicates (Figure 2).

**FIGURE 2:**
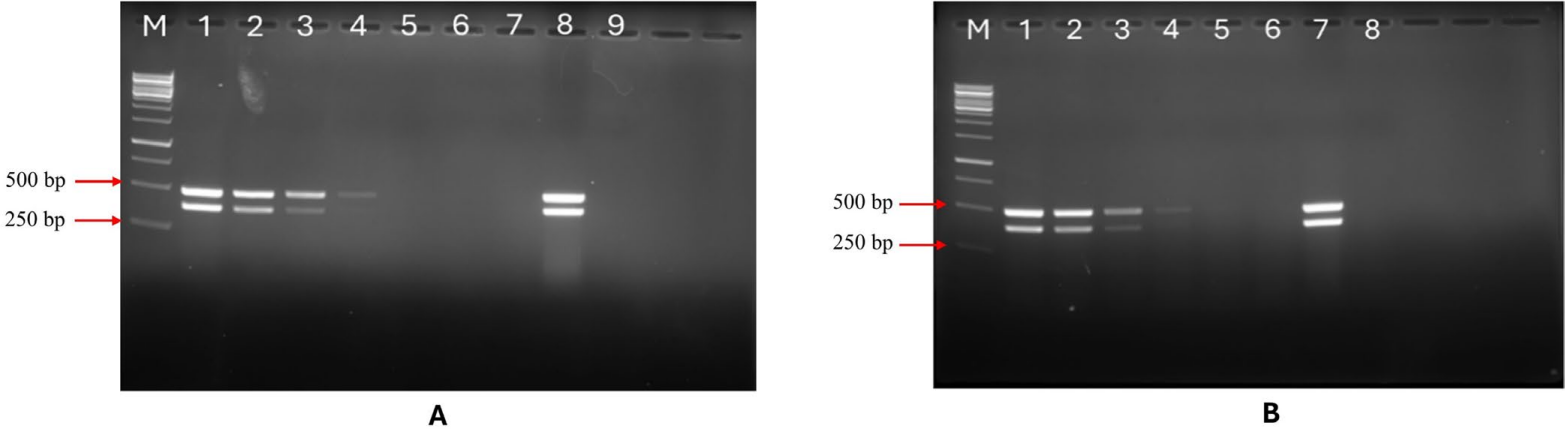
Limit of detection of the duplex PCR targeting potentially pathogenic *Vibrio harveyi* . Agarose gel electrophoresis of the PCR products obtained from DNA extracted from 10‐fold serial dilutions of an overnight culture of *V. harveyi* C2 (A) and from DNA extracted from sea bass splenic tissue inoculated with 10‐fold serial dilutions of an overnight culture of *V. harveyi* C2 (B). From left to right: (A) 1 kb molecular weight DNA ladder (M), 10^8^ CFU/mL (1), 10^7^ CFU/mL (2), 10^6^ CFU/mL (3), 10^5^ CFU/mL (4), 10^4^ CFU/mL (5), 10^3^ CFU/mL (6), 10^2^ CFU/mL (7), positive control ( *V. harveyi* C2) (8), negative control (9). (B) M, 10^8^ CFU/g (1), 10^7^ CFU/g (2), 10^6^ CFU/g (3), 10^5^ CFU/g (4), 10^4^ CFU/g (5), 10^3^ CFU/g (6), positive control ( *V. harveyi* C2) (7), negative control (8).

To improve assay sensitivity, an enrichment step consisting of incubation in APW‐1 for 8 h was incorporated into the protocol. Following enrichment, LOD was markedly reduced, reaching approximately 10^2^ CFU/g or mL in both water and fish tissue samples, as determined by reproducible amplification in all replicates (Figure 3 and data not shown). Lower bacterial concentrations (10 CFU/g) did not yield consistent amplification and were therefore not considered within the LOD. Together, these results demonstrate that the inclusion of a short enrichment step substantially enhances the sensitivity of the duplex PCR while maintaining high specificity.

**FIGURE 3:**
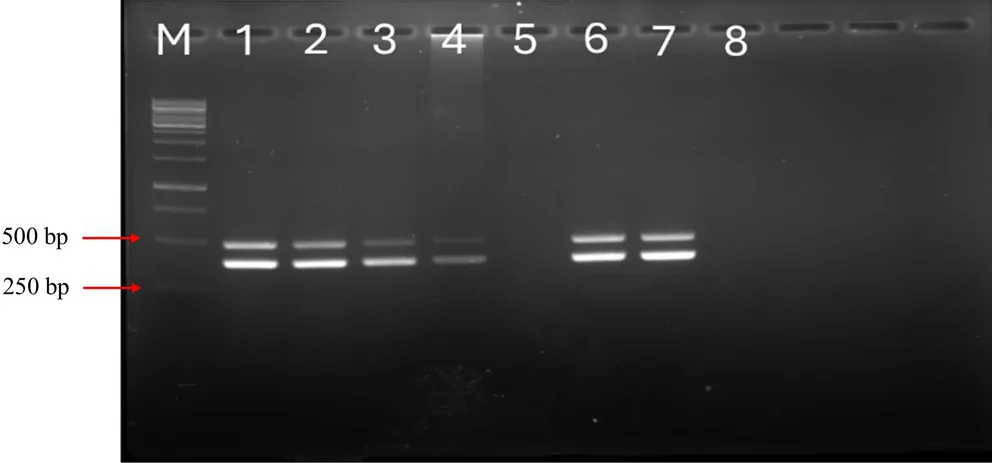
Limit of detection of the duplex PCR targeting potentially pathogenic *Vibrio harveyi* after sample enrichment. Agarose gel electrophoresis of the PCR products obtained from DNA extracted from sea bass splenic tissue samples inoculated with 10‐fold serial dilutions of an overnight culture of *V. harveyi* C2 and enriched for 8 h in alkaline peptone water with 1% NaCl. From left to right: 1 kb molecular weight DNA ladder (M), 10^5^ CFU/g (1), 10^4^ CFU/g (2), 10^3^ CFU/g (3), 10^2^ CFU/g (4), 10 CFU/g (5), positive control ( *V. harveyi* C2) (6), positive control (10^8^ CFU/g) (7), negative control (8).

### 3.2 Diagnostic Performance Evaluation

The performance of the two‐step duplex PCR protocol was evaluated using both experimentally inoculated samples and field samples representative of aquaculture and surrounding marine environments. Two DNA extraction methods, a commercial genomic DNA purification kit and a modified boiling technique, were initially compared during evaluation. Both methods yielded DNA of comparable quality, with *A*_260_/*A*_280_ ratios ranging from 1.7 to 2.0 and produced equivalent PCR results. Based on these findings, the boiling technique was selected for subsequent analyses due to its simplicity, rapidity and cost‐effectiveness.

Analytical performance was assessed using fish splenic tissue artificially inoculated with known concentrations of *V. harveyi* strain C2. Without enrichment, duplex PCR consistently detected both target markers only at bacterial concentrations equal to or above 10^5^ CFU/g. Following the incorporation of an 8 h enrichment step in APW‐1, reliable detection was achieved at concentrations as low as 10^2^ CFU/g, confirming the sensitivity improvement observed during analytical LOD determination. These results demonstrate the robustness of the protocol when applied to complex biological matrices.

Field evaluation was performed using environmental water samples and tissues collected from apparently healthy European sea bass. Water samples collected from La Albufera lagoon and a nearby Mediterranean beach were subjected to enrichment prior to analysis. Duplex PCR detected *V. harveyi* (*toxR*‐positive) exclusively in samples with a salinity of 0.5%, whereas no amplification was observed in water samples with lower salinity (Figure 4A). None of the environmental water samples tested positive for the *fpcrp* marker. In parallel, microbiological culture confirmed the presence of *V. harveyi* isolates lacking the *fpcrp* gene, in agreement with PCR results.

**FIGURE 4:**
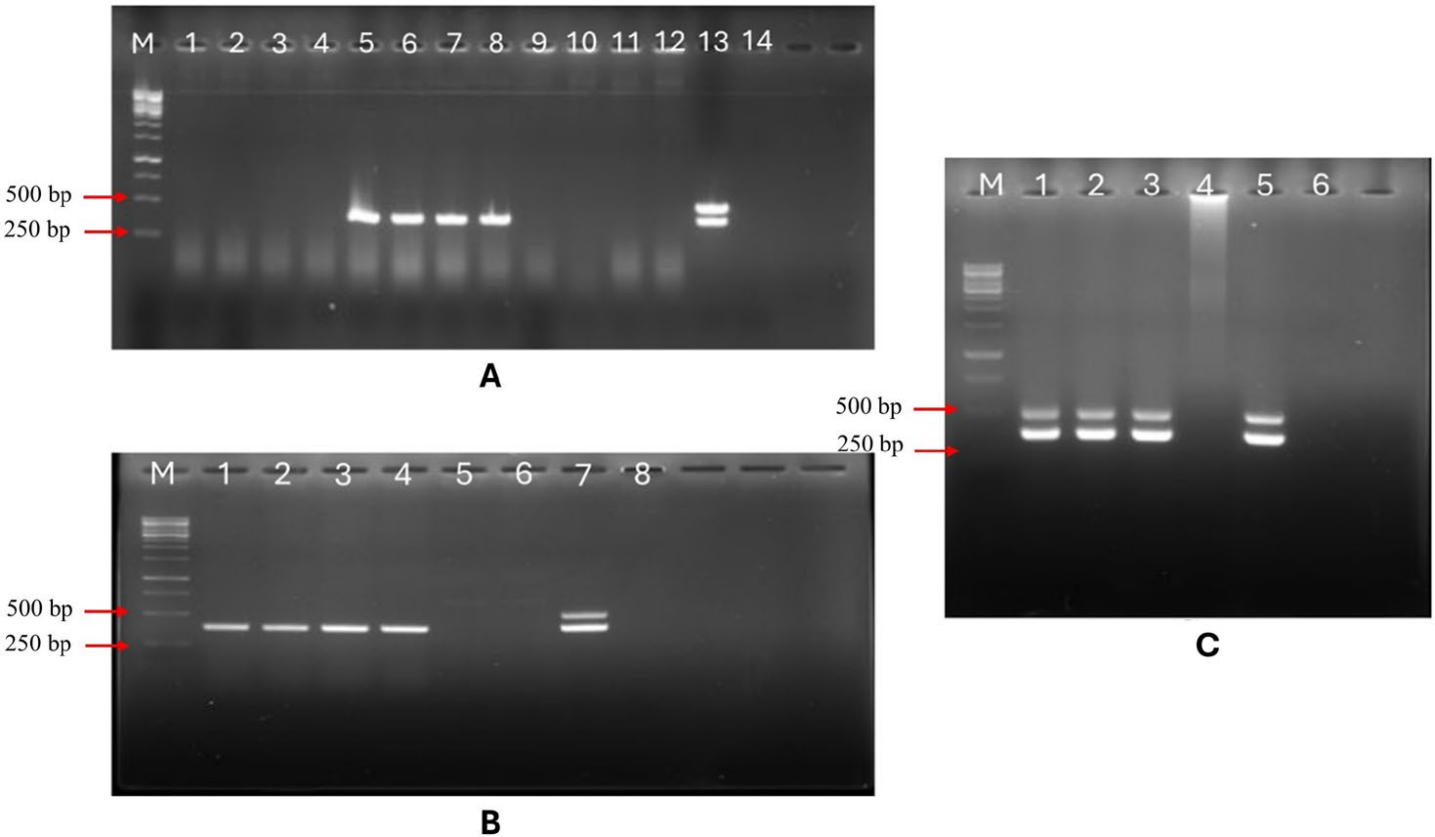
Evaluation of diagnostic performance of the two‐step PCR protocol targeting potentially pathogenic *Vibrio harveyi* with environmental samples. Agarose gel electrophoresis of the PCR products obtained from the DNA extracted from environmental samples (A) and healthy seabass tissues sampled in spring (B) and autumn (C). (A) Lanes from 1 to 12 correspond to DNA extracted from water sampled in different points in La Albufera lagoon and nearby beach, followed by the positive control ( *V. harveyi* C2) (13) and the negative control (14). (B) Lanes correspond to DNA extracted from skin mucus (1, 2), gills (3–4) and blood (5–6); positive control ( *V. harveyi* C2) (7); and negative control (8). (C) Lanes correspond to DNA extracted from gills (1–3) and blood (4), positive control *V. harveyi* C2 (5) and negative control (6). 1 kb molecular weight DNA ladder (Lane M).

Fish tissue samples were analysed from two independent sampling events. In the first sampling, *V. harveyi* (*toxR*‐positive) was detected in gill and skin mucus samples, while all samples were negative for *fpcrp* amplification (Figure 4B). Culture‐based analysis corroborated these results, yielding *V. harveyi* isolates lacking the putative virulence‐associated marker. In contrast, during the second sampling event, duplex PCR identified isolates positive for both *toxR* and *fpcrp* in gill tissue (Figure 4C). Microbiological culture confirmed the presence of *V. harveyi* isolates harbouring *fpcrp* gene, in agreement with PCR results.

Overall, these diagnostic performance results demonstrate that the two‐step duplex PCR protocol is suitable for the detection of *V. harveyi* in both environmental and host‐associated samples and is capable of discriminating strains with different virulence potential under field‐relevant conditions.

## 4 Discussion

In this study, we developed and optimised an enrichment‐based duplex PCR protocol that enables reliable detection of *V. harveyi* (targeting the species‐specific *toxR* gene) and discrimination of strains with increased virulence potential (targeting the plasmid‐associated *fpcrp* gene) under field‐relevant conditions. The *fpcrp* gene was only detected in ‘virulent’ *V. harveyi* genomes, supporting the choice of this gene as a putative virulence‐associated marker to be incorporated in the novel PCR assay.

Rather than relying on PCR alone, the proposed approach combines a short selective enrichment step with molecular detection, which proved essential for effective application in complex biological and environmental samples where *V. harveyi* is often present at low abundance and within diverse microbial communities (Mougin et al. 2021).

Direct application of the duplex PCR to fish tissues and water samples showed limited sensitivity, with a detection threshold of approximately 10^5^ CFU/g or mL. While this performance is acceptable for heavily infected samples, it is insufficient for early‐stage surveillance or environmental monitoring in aquaculture settings, where low bacterial loads may precede disease outbreaks or reflect subclinical persistence (Mougin et al. 2021). The incorporation of an 8 h enrichment step in APW‐1 markedly enhanced sensitivity, lowering the LOD to approximately 10^2^ CFU/g or mL without compromising specificity. These results demonstrate that the effectiveness of the method in field samples relies on the combined enrichment–PCR strategy, rather than on the molecular assay alone.

This finding is particularly relevant considering the ecological behaviour of *V. harveyi*. Like other members of the genus *Vibrio*, *V. harveyi* can enter a viable but non‐culturable (VBNC) state under unfavourable environmental conditions, in which cells remain viable but display extremely low metabolic activity and lose culturability, preventing detection by conventional culture‐based methods (Ramaiah et al. 2002). Upon return to favourable conditions, VBNC cells may resuscitate and regain culturability, increasing the risk of unexpected disease outbreaks. In addition, in natural aquatic environments *V. harveyi* often represents a minor fraction of the microbial community, further hampering direct detection (Montánchez and Kaberdin 2020). The enrichment step included in the present protocol favours the growth of *V. harveyi* cells present at low abundance, thereby enabling their subsequent molecular detection in complex matrices.

Horizontal gene transfer (HGT) plays a central role in the ecology and evolution of vibrios and is a major driver of the emergence of highly virulent fish‐pathogenic clones (Hazen et al. 2010). This is well documented in *V. vulnificus* , where acquisition of plasmid‐encoded virulence genes is a key determinant of pathogenicity (Amaro et al. 2020). Homologous virulence‐associated plasmids and genes have recently been identified in *V. harveyi* , most likely acquired through HGT from *V. vulnificus* or other bacterial species coexisting in aquaculture environments (Carmona‐Salido et al. 2021). Moreover, specific *V. harveyi* plasmids have recently been demonstrated to act as key drivers of virulence in marine fish hosts such as barramundi (Sullivan et al. 2025).

The *fpcrp* gene represents a highly conserved putative virulence‐associated marker (Hernández‐Cabanyero et al. 2019). Its high sequence conservation in positive strains suggests a conserved biological function and possibly a recent horizontal transfer event. However, further functional characterisation, including gene‐knockout mutagenesis and in vivo challenge assays, will be required to determine its precise contribution to the pathogenicity of *V. harveyi* strains harbouring this gene.

The duplex PCR assay developed here exploits this biological feature by combining a species‐specific marker (*toxR*) with the virulence‐associated *fpcrp* gene. Analytical specificity assays confirmed that *toxR* amplification was restricted to *V. harveyi* , with no cross‐reactivity detected for other closely related *Vibrio* species commonly found in aquaculture environments.

Interpretation of the *fpcrp* marker exclusively in *toxR*‐positive samples ensured unambiguous attribution of virulence potential to *V. harveyi* , even in mixed bacterial populations. This dual‐target strategy provides a significant advantage over existing diagnostic methods, which generally allow species identification but do not discriminate between potentially virulent and non‐virulent strains.

Field evaluation of the complete protocol revealed an informative distribution of *V. harveyi* isolates with respect to virulence potential. *V. harveyi* isolates lacking the *fpcrp* marker were detected and isolated from environmental water samples as well as from fish skin mucus and gill surfaces, indicating that *V. harveyi* isolates without the putative virulence‐associated marker circulate in both environmental and host‐associated niches. In contrast, *fpcrp*‐positive isolates were detected only in fish surface samples during one of the sampling events. Importantly, the sampled fish did not exhibit clinical signs of vibriosis and had been previously vaccinated against *V. harveyi* . This is consistent with the interpretation that vaccination and a properly functioning immune system can effectively restrain pathogen proliferation and prevent disease, even in the presence of potentially virulent strains. Under such conditions, virulent *V. harveyi* clones may persist at mucosal sites without causing overt pathology. These findings highlight the value of surveillance tools capable of detecting putative virulence‐associated markers before environmental stressors or host impairment compromise immune control and trigger disease outbreaks.

Environmental parameters also influenced *V. harveyi* detection in natural samples. In agreement with previous studies, salinity emerged as a decisive factor for the survival and persistence of this species (Zhang et al. 2020), with detection restricted to samples exhibiting salinity values of approximately 0.5% or higher (Barriga‐Cuartero et al. 2024). The absence of *V. harveyi* in freshwater samples further supports the ecological relevance of salinity thresholds when interpreting environmental surveillance data.

A major strength of the proposed protocol lies in the incorporation of the *fpcrp* gene as a putative diagnostic target associated with specific pathogenic lineages within an enrichment‐based detection framework. This approach goes beyond simple pathogen detection and provides a risk‐oriented assessment by identifying strains with increased virulence potential. Such information is particularly valuable for aquaculture surveillance, early warning systems and disease prevention strategies, especially in the context of climate change, where rising seawater temperatures are known to favour the emergence and spread of *V. harveyi* ‐associated infections (Montánchez and Kaberdin 2020).

Overall, the combination of a short enrichment step with duplex PCR establishes a robust, sensitive and operationally practical diagnostic tool that balances performance, speed and cost‐effectiveness. Compared with conventional culture‐based diagnostics or single‐target molecular assays, the proposed protocol would be well suited for routine monitoring in aquaculture facilities and surrounding environments. However, further studies are needed (e.g., with larger samples and inter‐laboratory reproducibility tests) before it can be considered validated for routine use.

Future work should focus on further optimisation of the method. In particular, shortening the enrichment step or adapting the assay to an isothermal amplification format, thereby eliminating the need for a thermocycler, would further enhance its applicability for rapid, on‐site diagnostics and continuous surveillance in aquaculture settings.

## 5 Conclusions

The enrichment‐based duplex PCR protocol developed in this study provides a specific, sensitive and operationally practical tool for the detection of *V. harveyi* strains with different virulence potential in marine aquaculture. By combining a short selective enrichment step with molecular detection of both a species‐specific marker (*toxR*) and a putative virulence‐associated gene (*fpcrp*), the protocol enables reliable identification of *V. harveyi* in complex biological and environmental matrices under field conditions.

Importantly, the inclusion of the enrichment step proved essential to achieve the sensitivity required for early detection and surveillance, particularly in samples where *V. harveyi* is present at low abundance or coexists with diverse microbial communities. This integrated approach allows risk‐oriented monitoring by discriminating potentially virulent isolates prior to the onset of clinical disease, thereby supporting timely implementation of biosecurity and disease prevention measures in aquaculture facilities.

Future studies should explore the application of this protocol across different aquaculture species and production systems, as well as its adaptation to automated, high‐throughput or isothermal amplification platforms. Such developments, combined with additional studies using larger sample sizes, would further increase the potential for its application in routine monitoring and on‐site diagnostics, contributing to improved management of *V. harveyi* infections in a changing environmental context.

## Author Contributions

**Javier Barriga‐Cuartero:** investigation, writing – original draft, methodology, writing – review and editing, formal analysis, data curation. **Carla Hernández‐Cabanyero:** writing – review and editing, methodology, formal analysis, data curation, investigation. **Pablo Ibanyez‐Paya:** investigation, writing – review and editing. **Carmen Amaro:** conceptualization, investigation, funding acquisition, validation, writing – review and editing, supervision. **Belen Fouz:** conceptualization, investigation, funding acquisition, writing – original draft, writing – review and editing, validation, supervision.

## Funding

This study forms part of the ThinkInAzul programme and was supported by the Ministerio de Ciencia, Innovación y Universidades (MCIU) with funding from the European Union NextGeneration EU (PRTR‐C17.I1), and by the Generalitat Valenciana (GVA‐THINKINAZUL/2021/027) (Principal Investigators: C. Amaro and B. Fouz, Universitat de València, UV). Additional support was provided by projects PID2020‐120619RB‐I00 and PID2024‐162627OB‐I00, funded by MCIN/AEI/10.13039/501100011033 and by MICIU/AEI/10.13039/501100011033 and FEDER, EU, respectively, as well as by AICO2022 (CIAICO/2021/293) and CIAICO/2024/236, both funded by the Conselleria de Educación, Cultura y Universidades (Generalitat Valenciana) J. Barriga‐Cuartero got funds from grant PRE2021‐099708 and P. Ibanyez‐Paya from grant MRR‐GVA Programa Investigo 2022.

## Ethics Statement

All assays involving the handling of fish during the study were approved by the Institutional Animal Care and Use Committee and the local authority (Consellería de Agricultura, Medio Ambiente, Cambio Climático y Desarrollo Rural. Generalitat Valenciana, project licence 2023/VSC/PEA/0141, 06.07.2023) and the Bioethic Committee of the Universitat de València, following the European Directive 2010/63/EU and the Spanish law ‘Real Decreto 53/2013’.

## Conflicts of Interest

The authors declare no conflicts of interest.

## Supporting information


**Figure S1:** Location of the fish farm (gulf of Valencia, Spain). Tissue samples were obtained from healthy European sea bass exemplars for the validation of the two‐step duplex PCR protocol. Two distinct sampling events were conducted in June and September of 2022, respectively.
**Figure S2:** Locations of the water sampling points in the La Albufera lagoon (Valencia, Spain) and nearby beach. Each numbered point (1–12) directly corresponds to the sample loaded (1–12) of the agarose gel shown in Figure 4A.
**Table S1:** Location and environmental characteristics of water sampling sites in ‘La Albufera’ (Valencia, Spain).

## Data Availability

The data that support the findings of this study are available from the corresponding author upon reasonable request.
