# Supplementary material for: Enrichment‐Based Duplex PCR for Early Detection of Potentially Virulent Vibrio harveyi in Aquaculture
Source: J Fish Dis. 2026 Jun 24;49(11):e70224. doi: 10.1111/jfd.70224 (PMC13629451; doi:10.1111/jfd.70224)
Supplement: Supplementary file 1 [file JFD-49-e70224-s001.docx]

**Supplementary data**

**Figure S1. Location of the fish farm (gulf of Valencia, Spain).** Tissue samples were obtained from healthy European sea bass exemplars for the validation of the two-step duplex PCR protocol. Two distinct sampling events were conducted in June and September of 2022, respectively.

**Figure S2. Locations of the water sampling points in the La Albufera lagoon (Valencia, Spain) and nearby beach.** Each numbered point (1-12) directly corresponds to the sample loaded (1-12) of the agarose gel shown in Figure 4A.

**Table S1. Location and environmental characteristics of water sampling sites in “La Albufera” (Valencia, Spain).**

| **Sampling site** | **Water temperature (ºC)** | **Water salinity (%)** | **Coordinates** |
| --- | --- | --- | --- |
| 1 | 30 | 0.2 | 39.349, -0’323 |
| 2 | 30 | 0.2 | 39.343, -0.322 |
| 3 | 30 | 0.2 | 39.332, -0.319 |
| 4 | 30 | 0.2 | 39.324, -0.344 |
| 5 | 29 | 0.5 | 39.348, -0.319 |
| 6 | 29 | 0.5 | 39.350, -0.312 |
| 7 | 29 | 0.5 | 39.308, -0.293 |
| 8 | 29 | 0.5 | 39.308, -0.291 |
| 9 | 30 | 0.2 | 39.316, -0.390 |
| 10 | 30 | 0.2 | 39.336, -0.386 |
| 11 | 30 | 0.2 | 39.343, -0.379 |
| 12 | 30 | 0.2 | 39.351, -0.364 |
